# Supplementary material for: Empirical methods for controlling false positives and estimating confidence in ChIP-Seq peaks
Source: BMC Bioinformatics. 2008 Dec 5;9:523. doi: 10.1186/1471-2105-9-523 (PMC2628906; doi:10.1186/1471-2105-9-523)
Supplement: Additional File 1 — A variety of html documents from the USeq web site detailing the available applications, their best usage, output file type descriptions, command line menus, etc. [file 1471-2105-9-523-S1.zip › USeqUserGuides/JavaDoc.zip!/JavaDoc/trans/main/WilcoxonSignedRankTest.html]

WilcoxonSignedRankTest


|  |  |  |
| --- | --- | --- |
| **Overview** \| **Package** \| **Class** \| **Use** \| **Tree** \| **Deprecated** \| **Index** \| **Help** | |  |
| **PREV CLASS**   **NEXT CLASS** | **FRAMES**    **NO FRAMES**     **All Classes** |  |
| SUMMARY: NESTED \| FIELD \| CONSTR \| METHOD | DETAIL: FIELD \| CONSTR \| METHOD |  |


---


## trans.main Class WilcoxonSignedRankTest

```
java.lang.Object
  trans.main.WilcoxonSignedRankTest
```

---

``` public class WilcoxonSignedRankTest extends java.lang.Object ```

Implementation of a Wilcoxon Signed Rank Test.
Many issues to consider: what to do about ties, what to do about zero diffs, what to use as the output
(WPlus, WMinus, WCombine, which z-value (Zw or Zs), a p-value, or Affys -10\*LogBase10(p-value) transformation,
lastly how to handle situations where N<10.
The minimal constructor here averages ties, throws out zero diffs, uses the Lowry z-value calculation method
and the Affy p-value transformation (transformedPValueFloat) as the final output.
Note: this transformedPValueFloat may be 0 indicating no significance (or N<5) (p-value > 0.05), 90 "very" significant (p-value < 1E-11)
or something measurable in between. Affys favored 10-5 p-value cutoff corresponds to a LogTrans= 49.4, with bigger numbers
"more significant" although in reality, anything bigger than LogTrans 13.1 is, by WSRT, significant, assuming a normal distribution
and >10 non zero difference sample pairs.
Uncomment the main() to test and tweak.

---

**Constructor Summary**

`WilcoxonSignedRankTest()`

`WilcoxonSignedRankTest(float[] treatment,
float[] control)`
  
          Minimal constructor, fastest way to calculate a transformedPValueFloat


| **Method Summary** | |
| --- | --- |
| `double` | `calcSmallSample10Log10PValue(float W, int N)`             For calculating a -10Log10(pValue) for small sample sizes (N 5-9). |
| `void` | `calculateSignedRankStatistics()`             Calculates signed rank statistic S = sum of the ranks (aka WPlus) for WilcoxonSamplePairs with a positive difference and WMinus for those with a negative difference. |
| `void` | `calculateTestStatisticZw()`             Calculates test statistic Z from Lowry . |
| `void` | `createSamplePairs(float[] treatment, float[] control, boolean noZeros)`             Creates WilcoxonSamplePair objects from two float[]s. |
| `double[]` | `getAverageIntensityValues()` |
| `int` | `getN()` |
| `int` | `getNumberWMinusRanks()` |
| `int` | `getNumberWPlusRanks()` |
| `double` | `getPValue()`             A p-value of -1 is the default insignificant number. |
| `double` | `getTransformedPValue()` |
| `java.lang.String` | `getTransformedPValueString()` |
| `float` | `getW()` |
| `float` | `getWMinus()` |
| `float` | `getWPlus()` |
| `WilcoxonSamplePair[]` | `getWsps()` |
| `float` | `getZw()` |
| `static void` | `main(java.lang.String[] args)` |
| `void` | `rankSamplePairs()`             Ranks a sorted array of WilcoxonSamplePairs based on absoluteDifference. |
| `void` | `setWsps(WilcoxonSamplePair[] pairs)` |
| `static float[]` | `signedDifference(float[] scores, float median)`             Returns signed difference between the scores and a median. |
| `double` | `test()` |
| `java.lang.String` | `toString()` |

**Methods inherited from class java.lang.Object**

`equals, getClass, hashCode, notify, notifyAll, wait, wait, wait`

**Constructor Detail**

### WilcoxonSignedRankTest

```
public WilcoxonSignedRankTest(float[] treatment,
                              float[] control)
```

:   Minimal constructor, fastest way to calculate a transformedPValueFloat

---


### WilcoxonSignedRankTest

```
public WilcoxonSignedRankTest()
```


**Method Detail**

### test

```
public double test()
```

---


### calculateTestStatisticZw

```
public void calculateTestStatisticZw()
```

:   Calculates test statistic Z from Lowry .

---


### calculateSignedRankStatistics

```
public void calculateSignedRankStatistics()
```

:   Calculates signed rank statistic S = sum of the ranks (aka WPlus) for WilcoxonSamplePairs with a
    positive difference and WMinus for those with a negative difference.

---


### rankSamplePairs

```
public void rankSamplePairs()
```

:   Ranks a sorted array of WilcoxonSamplePairs based on absoluteDifference.
    If no ties are found then this is simply their array index number+1.
    (ie 1,2,3,4...)
    If ties are encountered, ties are assigned the average of their index
    positions+1. (ie if index+1's: 2,3,4 have the same absolute difference, all are assigned a
    rank of 3).

---


### createSamplePairs

```
public void createSamplePairs(float[] treatment,
                              float[] control,
                              boolean noZeros)
```

:   Creates WilcoxonSamplePair objects from two float[]s.
    Indicate if you want to throw out pairs with a difference of zero.
    (true for no zeros, false for keep zeros

---


### signedDifference

```
public static float[] signedDifference(float[] scores,
                                       float median)
```

:   Returns signed difference between the scores and a median.

---


### calcSmallSample10Log10PValue

```
public double calcSmallSample10Log10PValue(float W,
                                           int N)
```

:   For calculating a -10Log10(pValue) for small sample sizes (N 5-9).

---


### toString

```
public java.lang.String toString()
```

:   **Overrides:**: `toString` in class `java.lang.Object`

---


### getWsps

```
public WilcoxonSamplePair[] getWsps()
```

---


### setWsps

```
public void setWsps(WilcoxonSamplePair[] pairs)
```

---


### getZw

```
public float getZw()
```

---


### getNumberWMinusRanks

```
public int getNumberWMinusRanks()
```

---


### getNumberWPlusRanks

```
public int getNumberWPlusRanks()
```

---


### getW

```
public float getW()
```

---


### getWMinus

```
public float getWMinus()
```

---


### getWPlus

```
public float getWPlus()
```

---


### getTransformedPValue

```
public double getTransformedPValue()
```

---


### getTransformedPValueString

```
public java.lang.String getTransformedPValueString()
```

---


### main

```
public static void main(java.lang.String[] args)
```

---


### getN

```
public int getN()
```

---


### getAverageIntensityValues

```
public double[] getAverageIntensityValues()
```

---


### getPValue

```
public double getPValue()
```

:   A p-value of -1 is the default insignificant number. Check the sample size, must have > 4 pairs to calculate wilcoxon.


---


|  |  |  |
| --- | --- | --- |
| **Overview** \| **Package** \| **Class** \| **Use** \| **Tree** \| **Deprecated** \| **Index** \| **Help** | |  |
| **PREV CLASS**   **NEXT CLASS** | **FRAMES**    **NO FRAMES**     **All Classes** |  |
| SUMMARY: NESTED \| FIELD \| CONSTR \| METHOD | DETAIL: FIELD \| CONSTR \| METHOD |  |


---
